# Evaluation of gafchromic EBT film for intensity modulated radiation therapy dose distribution verification

**DOI:** 10.4103/0971-6203.26693

**Published:** 2006

**Authors:** A. Sankar, P. G. Goplakrishna Kurup, V. Murali, Komanduri M. Ayyangar, R. Mothilal Nehru, J. Velmurugan

**Affiliations:** a Department of Radiotherapy, Apollo Speciality Hospital, Chennai, India; b Department of Radiotherapy, University of Nebraska Medical Center, Omaha, US; c Department of Physics, Anna University, Chennai, India

**Keywords:** EDR2 film, intensity modulated radiotherapy, radiochromic film

## Abstract

This work was undertaken with the intention of investigating the possibility of clinical use of commercially available self-developing radiochromic film – Gafchromic EBT film – for IMRT dose verification. The dose response curves were generated for the films using VXR-16 film scanner. The results obtained with EBT films were compared with the results of Kodak EDR2 films. It was found that the EBT film has a linear response between the dose ranges of 0 and 600 cGy. The dose-related characteristics of the EBT film, like post-irradiation color growth with time, film uniformity and effect of scanning orientation, were studied. There is up to 8.6% increase in the color density between 2 and 40 h after irradiation. There was a considerable variation, up to 8.5%, in the film uniformity over its sensitive region. The quantitative difference between calculated and measured dose distributions was analyzed using Gamma index with the tolerance of 3% dose difference and 3 mm distance agreement. EDR2 films showed good and consistent results with the calculated dose distribution, whereas the results obtained using EBT were inconsistent. The variation in the film uniformity limits the use of EBT film for conventional large field IMRT verification. For IMRT of smaller field size (4.5 × 4.5 cm), the results obtained with EBT were comparable with results of EDR2 films.

The quantitative dose validation of intensity modulated radiotherapy (IMRT) requires the three-dimensional high-resolution dosimetry systems with uniform sensitivity over its entire sensitive region. Silver halide radiographic films offer good resolution for two-dimensional dosimetry. Three-dimensional verification can be accomplished by measuring at different planes using films. The spectral sensitivity and response to the developing conditions are the important limiting factors of the silver halide films.[1] Self-developing radiochromic films offer high-resolution dose measurements with relatively insensitive spectral response.[2] Since these films are self-developing, the uncertainties associated with the developing conditions are ruled out. The present study deals with the evaluation of new type of radiochromic films – Gafchromic external beam therapy (EBT) film – for its use of IMRT plan validation. The results obtained with EBT films are compared with the results of commonly used Kodak extended dose range 2 (EDR2) films.

## Materials and Methods

### Film calibration

**Figure 1:**
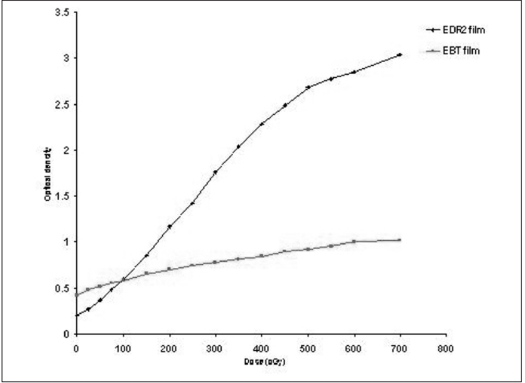
Sensitometric response of EDR2 and EBT films

The calibration data set for both EDR2 and EBT films were obtained by exposing the films with doses ranging from 19.6 cGy to 700 cGy using 6MV photon beams. The film was kept at a dmax in solid water phantom in a plane perpendicular to the beam axis. The processed EDR2 films were scanned using VIDAR 16 (VXR-16) digitizer and RIT 113 film dosimetry software. The irradiated EBT films were scanned 3 h after irradiation along portrait direction to allow the saturation of color growth, as recommended by the film manufacturer. The EBT films are scanned with the yellow filter supplied by the film manufacturer to enhance the sensitivity of the film with VXR 16 scanner as the EBT film strongly absorbs the higher wavelength region of the visible spectrum. The measured optical density (OD) was plotted as a function dose [Figure 1]. From the plot, it is evident that for the EDR2 films, the OD ranges from 0.204 to 3.04 for the dose range of 0 to 700 cGy with the linear response up to 500 cGy. In contrast, an EBT film shows a narrow OD range of 0.42 to 1.02.

### Post-irradiation color growth of EBT films

**Figure 2:**
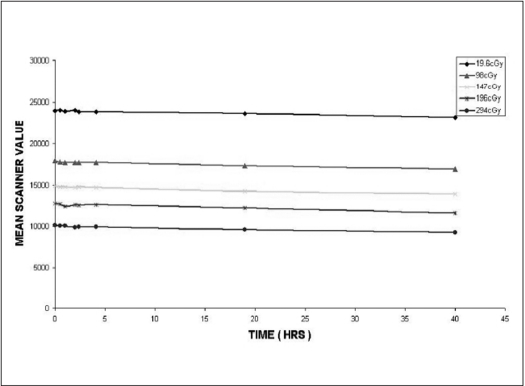
Variation in Mean scanner value as a function of time for different doses

Many authors (Chu *et al,*[3] Muench *et al*[4] and Meigooni *et al*[5]) have reported the color density growth of previous models of Gafchromic films. To determine the time dependence of change in color density of irradiated EBT film, five pieces of EBT film were irradiated to the doses of 19.6 cGy, 98 cGy, 147 cGy, 196 cGy and 294 cGy respectively. The optical densities of these films were measured at different times after irradiation for about 40 h. The scanner value measured at different times after irradiation for above-mentioned doses is plotted in Figure 2. From the plot, it is evident that the scanner value decreases as a function of time. The percentage of increase in density between 2 and 40 h after irradiation is tabulated in Table 1.

**Table 1:** Variation in optical density between 2 and 40 h after irradiation

*Dose (cGy)*	*% of increase in OD*
19.6	3.44
98	4.73
147	5.75
196	8.62
294	6.71

### Film uniformity

**Figure 3:**
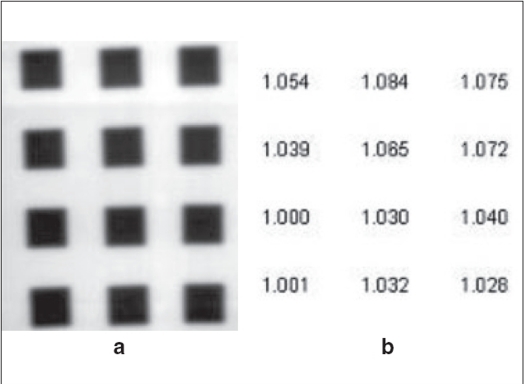
EBT film uniformity check, a: Irradiated film, b: Relative OD values

Previous models of radiochromic films have showed a large variation in uniformity, particularly along the coating direction.[6–8] To study the spatial uniformity of the EBT film, the film was irradiated by 3 × 3 cm field at 12 different places (a 3 × 4 matrix) for equal dose of 150 cGy. The film was scanned with the filter 24 h after irradiation. All other experimental films were scanned 2 h after irradiation, but this film was scanned 24 h after irradiation to allow for the maximum color growth. The irradiated film and relative values of OD, normalized to minimum OD measured, corresponding to irradiated positions are shown in Figure 3. From the figure, it was evident that the film uniformity varied up to 8.5% between two regions of the film.

### Effect of scanning direction on sensitometry of EBT film

**Figure 4:**
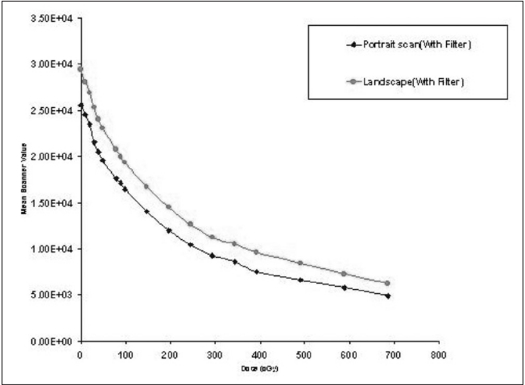
Sensitometric response of film with respect to scanning orientation

To study the effect of scanning direction on scanner value, the irradiated EBT films were scanned along both portrait and landscape directions. The mean scanner value was plotted against dose for both the scans [Figure 4]. It is evident from the plot that the overall scanner values for landscape direction scanning seem to be higher than that of portrait scanning for the same film and dose. The explanation given by the manufacturer for this is the anisotropic scattering of transmitted light by the needle-like active components, which are aligned in a direction of smaller dimension of the film.

### IMRT dose distribution verification

The IMRT plans generated by Brainscan planning system were verified using EDR2 and EBT films. For the axial dose comparison, hybrid plans of patient plans were generated on scanned images of Med-Tec IMRT phantom. The EDR2 and EBT films were sandwiched together and irradiated simultaneously for plan verification. The intensity pattern verifications were performed by irradiating the films in a plane perpendicular to the beam axis at a depth of 1.5 cm in a solid water phantom. Scanned films were calibrated using their respective calibration data set. The fluence and dose distribution patterns corresponding to the film positions in the phantoms were derived from respective planning systems. The film and calculated dose pattern are co-registered for analysis. Using the template registration methods in RIT113 software, the plan-film fiducial points and their respective template points were registered unambiguously. The qualitative comparisons like overlaid isodoses [Figures 5a and 5b] and profiles [Figures 6a and 6b] and quantitative comparisons like Gamma index with a tolerance of 3% dose difference and 3 mm distance agreement were performed to check the consistency between calculated and measured dose distributions [Table 2].

**Figure 5a:**
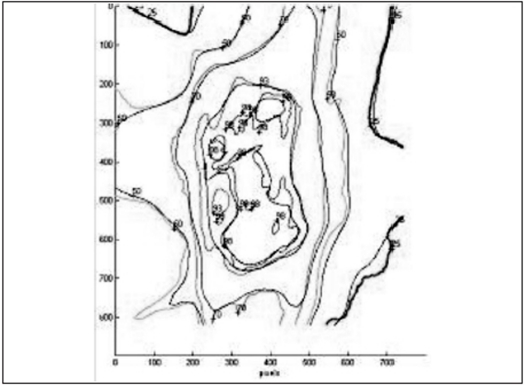
Comparison of EDR2 dose distribution (thick lines) over the IMRT plan (thin lines)

**Figure 5b:**
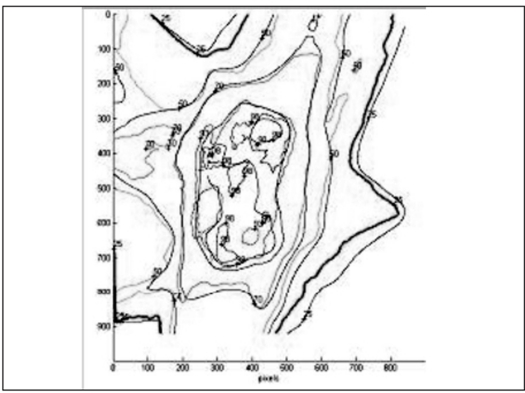
Comparison of EBT dose distribution (thick lines) over the IMRT plan (thin lines)

**Figure 6a:**
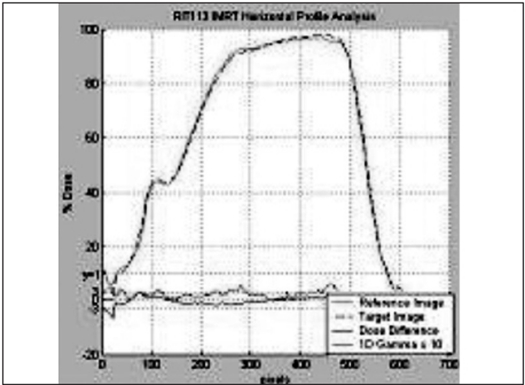
Comparison of EDR2 profile (solid line) over the IMRT plan (dotted lines)

**Figure 6b:**
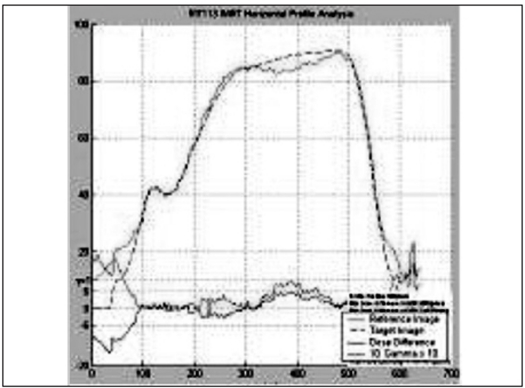
Comparison of EBT profile (solid line) over the IMRT plan (dotted lines)

**Table 2:** Results of mean Gamma values for the selected IMRT studies with EDR2 and EBT films

*Study*	*No. of pixels*		*Mean Gamma*		*Std deviation*		*Percentage of pixels passing Gamma*	
				
	*EDR*	*EBT*	*EDR*	*EBT*	*EDR*	*EBT*	*EDR*	*EBT*
Study1	511924	470600	0.437	0.797	0.329	0.581	93.36	69.55
Study2	336968	327756	0.284	0.599	0.235	2.010	98.97	84.26
Study3	277632	277672	0.418	0.542	0.310	0.671	96.30	85.14
Study4	324611	248292	0.358	1.005	0.406	1.130	93.31	69.20
Study5	333670	314976	0.276	0.706	0.183	0.748	99.77	75.33
Study6	158323	145140	0.402	0.509	0.245	0.440	99.82	83.52
Study7	120827	116974	0.397	0.230	0.161	0.192	100.00	99.89
Study8	244240	247127	0.248	0.555	0.183	0.522	99.93	78.45
Study9	121312	114660	0.352	0.776	0.220	0.682	98.86	72.77
Study10	172872	165664	0.352	0.543	0.204	0.406	99.91	89.66
Study11	274560	200288	0.279	1.041	0.193	1.266	99.67	68.11
Study12	156825	149968	0.378	0.790	0.471	0.915	92.60	73.23
Study13	69958	66515	0.310	0.313	0.244	0.234	97.74	98.20
Study14	328497	327398	0.196	0.513	0.173	0.394	99.87	90.70
Study15	318642	314426	0.291	0.721	0.198	0.542	97.52	85.33

## Results and Conclusions

The Gafchromic EBT film has a better sensitivity when compared to the previous versions of Gafchromic films and that falls in the clinical dose range. Though EBT films respond with the narrow OD range, due to the high resolving power of VXR16 digitizing system, over 65,000 gray shades can be identified and about 50,000 fall within the OD of 1; the small variations in OD produced by the EBT films are interpretable. There is a significant growth in density even 2 h after irradiation; generally the percentage of growth in density increases with time and dose. The dose measurements of films scanned at different times after irradiation will affect the accuracy of measurements because of this differential density growth.

One of the following three methods can eliminate the effect of density growth on the measurement:

- By applying appropriate growth correction factors for individual doses
- By maintaining the same time difference between the film irradiation and scanning for both calibration and experimental films
- Creating a number of calibration data sets by scanning the calibration films at different times after irradiation so that appropriate calibration data set can be used depending on the scanning of experimental film after irradiation.

In our study, we have maintained the same time difference between the film irradiation and scanning for both calibration and experimental film to eliminate the effect of density growth.

For the same film and dose, the scanner value varies with the scanning direction of the EBT film. According to the explanation given by the manufacturer, this is due to the anisotropic transmission of light by the active components present in the coating of EBT film. The active component of the EBT film is a tiny needle-like structure. Because of their shape, they tend to align along their long axes parallel to the coating direction of the film. Normally, the coatings are performed in a direction parallel to the smaller (8”) side of the film. Because of the shape and alignment of the active components, relatively more light is scattered normal to the direction of coating than parallel to the direction of coating. It is necessary to scan all the calibration and experiment film in a same orientation and it is necessary to have a track on film orientation if it has been cut into pieces. Since the portrait scanning shows higher sensitivity of the film, it is recommended to perform portrait scanning.

In 15 dose comparisons done with both EDR2 and EBT films, EDR2 films show good and consistent agreement between measured and calculated dose distributions. The quantitative analysis between the calculated and measured dose distribution was evaluated using Gamma function. The mean Gamma values and percentage of pixels exceeding the tolerance are shown in Tables 2. From the tabulated data, it was evident that EDR2 films showed consistent and good agreement between the calculated and measured dose distribution, whereas with the EBT films, the results obtained were very inconsistent. For two specific studies (Study 7 and 13), the results obtained with EBT films were comparable with the results obtained with EDR2 films. But for other studies, the mean gamma and the percentage of pixels exceeding the tolerance values varied considerably when compared to the results obtained using EDR2 films. Better results were obtained for those two studies because of relatively smaller area of dose distribution (4.5 × 4.5 cm) and smaller number of pixels analyzed when compared to other studies. This strongly suggests that the variation in the spatial uniformity of the EBT film led to the large variation in the dose verification. For dose distributions of smaller area, better results can be achieved because over a small area, the film uniformity does not vary heavily. The measurement accuracy for the conventional large field dose distributions can be improved using double-irradiation method by delivering unknown dose followed by the delivery of known uniform dose to get the uniformity correction matrix of the experimental film. But it is a complex task; moreover, at present no software tool is commercially available to apply the uniformity correction matrix on the experimental film and perform the dose comparisons. So the inference from our study is that EBT film can be used for small field IMRT verification rather than the large field IMRT under present conditions.

## References

[1] Burch SE, Keafortt KJ, Trueblood JH, Sheils WC, Yeo JI, Yang CK (1997). A new approach to film dosimetry for high energy photon beams: Lateral scatter filtering. Med Phys.

[2] Niroomand A, Blackwell CR, Coursey BM, Gall KP, Galvin JM, McLaughlin WL (1998). Radiochromic film dosimetry: Recommendations of AAPM Radiation Therapy Committee Task Group 55. Med Phys.

[3] Chu RD, Van Dyke G, Lewis DF, O'Hara KP, Buckland BR, Dinelle F (1990). Gaf-chromic dosimetry media: A new high dose rate thin film routine dosimeter and dose mapping tool. Radiat Phys Chem.

[4] Meigooni AS, Sanders MF, Ibbott GS, Szeglin SR (1996). Dosimetry Characteristics of an improved radiochromic film. Med Phys.

[5] Muench PJ, Meigooni AS, Nath R, McLaughlin WL (1991). Photo energy dependence of the sensitivity of radiochromic film and comparison with silver halide film and LiF TLD's used for brachytherapy dosimetry. Med Phys.

[6] Mishra V, Williamson JF, Li Z, Meigooni AS (1993). Radiochromic film densitometry for Ir-192: Accuracy and precision. Med Phys.

[7] Meigooni AS, Klevin EE, Mishra V, Low DA, Purdy JA (1993). Gafchromic film dosimetry for megavoltage X-ray beams. Med Phys.

[8] Zhu Y, Kirov AS, Mishra V, Meigooni AS, Williamson JF (1995). Quantitative evaluation of radiochromic film response for two-dimensional dosimetry. Med Phys.

